# Supplementary material for: Effects of Allogeneic Mesenchymal Stem Cell Transplantation in Dogs with Inflammatory Bowel Disease Treated with and without Corticosteroids
Source: Animals (Basel). 2021 Jul 10;11(7):2061. doi: 10.3390/ani11072061 (PMC8300310; doi:10.3390/ani11072061)
Supplement: Supplementary file 1 [file animals-11-02061-s001.zip › animals-1254244-supplementary.pdf]

animals

MDPI

Article

Effects of Allogeneic Mesenchymal Stem Cell Transplantation in Dogs with Inflammatory Bowel Disease Treated with and without Corticosteroids.

José Ignacio Cristóbal [1,*], Francisco Javier Duque [1], Jesús Usón [1], Esther López [2], Patricia Ruiz [1] and Eva María Pérez [1]

Table S1. Significance level (p-value) between the values of each studied parameter (CIBDAI, CCECAI, albumin and cobalamin concentration) obtained at each review (T0, T1, T3, T6, and T12) in the MSC and P-MSC groups.

	CIBDAI		CCECAI		ALBUMIN		COBALAMIN	
	**MSC**	**P-MSC**	**MSC**	**P-MSC**	**MSC**	**P-MSC**	**MSC**	**P-MSC**
**T0-T1**	$p < 0.001$	$p < 0.001$	$p < 0.001$	$p < 0.001$	$p = 0.892$	$p = 0.880$	$p = 0.949$	$p = 0.998$
**T0-T3**	$p < 0.001$	$p < 0.001$	$p < 0.001$	$p < 0.001$	$p = 0.316$	$p = 0.179$	$p = 0.432$	$p = 0.891$
**T0-T6**	$p < 0.001$	$p < 0.001$	$p < 0.001$	$p < 0.001$	$p = 0.183$	$p = 0.048$	$p = 0.019$	$p = 0.975$
**T0-T12**	$p < 0.001$	$p < 0.001$	$p < 0.001$	$p < 0.001$	$p = 0.190$	$p = 0.045$	$p < 0.001$	$p = 0.933$
**T1-T3**	$p = 0.995$	$p = 0.520$	$p = 0.944$	$p = 0.523$	$p = 0.858$	$p = 0.651$	$p = 0.883$	$p = 0.968$
**T1-T6**	$p = 0.998$	$p = 0.290$	$p = 0.999$	$p = 0.216$	$p = 0.672$	$p = 0.284$	$p = 0.173$	$p = 0.996$
**T1-T12**	$p = 0.794$	$p = 0.162$	$p = 0.739$	$p = 0.131$	$p = 0.588$	$p = 0.216$	$p < 0.001$	$p = 0.982$
**T3-T6**	$p = 1.000$	$p = 0.986$	$p = 0.993$	$p = 0.962$	$p = 0.996$	$p = 0.968$	$p = 0.710$	$p = 1.000$
**T3-T12**	$p = 0.940$	$p = 0.896$	$p = 0.993$	$p = 0.854$	$p = 0.966$	$p = 0.864$	$p = 0.008$	$p = 1.000$
**T6-T12**	$p = 0.961$	$p = 0.995$	$p = 0.920$	$p = 0.997$	$p = 0.998$	$p = 0.994$	$p = 0.186$	$p = 1.000$
